# Characterization of marine diatom-infecting virus promoters in the model diatom *Phaeodactylum tricornutum*

**DOI:** 10.1038/srep18708

**Published:** 2015-12-22

**Authors:** Takashi Kadono, Arisa Miyagawa-Yamaguchi, Nozomu Kira, Yuji Tomaru, Takuma Okami, Takamichi Yoshimatsu, Liyuan Hou, Takeshi Ohama, Kazunari Fukunaga, Masanori Okauchi, Haruo Yamaguchi, Kohei Ohnishi, Angela Falciatore, Masao Adachi

**Affiliations:** 1 Laboratory of Aquatic Environmental Science (LAQUES), Faculty of Agriculture, Kochi University, Otsu-200, Monobe, Nankoku, Kochi 783-8502, Japan; 2 The United Graduate School of Agricultural Sciences, Ehime University, 3-5-7 Tarumi, Matsuyama, Ehime, 790-8566 Japan; 3 National Research Institute of Fisheries and Environment of Inland Sea, Fisheries Research Agency, 2-17-5 Maruishi, Hatsukaichi, Hiroshima 739-0452, Japan; 4 School of Environmental Science and Engineering, Kochi University of Technology, Tosayamada, Kami, Kochi 782-8502, Japan; 5 National Research Institute of Aquaculture, Fisheries Research Agency, 422-1 Nakatsuhamaura, Minami-ise, Mie 516-0193, Japan; 6 Research Institute of Molecular Genetics, Kochi University, Otsu-200, Nankoku, Kochi 783-8502, Japan; 7 Sorbonne Universités, UPMC Univ Paris 06, Institut de Biologie Paris-Seine, UMR 7238, F-75006 Paris, France; CNRS, UMR 7238, F-75006 Paris, France

## Abstract

Viruses are considered key players in phytoplankton population control in oceans. However, mechanisms that control viral gene expression in prominent microalgae such as diatoms remain largely unknown. In this study, potential promoter regions isolated from several marine diatom-infecting viruses (DIVs) were linked to the *egfp* reporter gene and transformed into the Pennales diatom *Phaeodactylum tricornutum*. We analysed their activity in cells grown under different conditions. Compared to diatom endogenous promoters, novel DIV promoter (ClP1) mediated a significantly higher degree of reporter transcription and translation. Stable expression levels were observed in transformants grown under both light and dark conditions, and high levels of expression were reported in cells in the stationary phase compared to the exponential phase of growth. Conserved motifs in the sequence of DIV promoters were also found. These results allow the identification of novel regulatory regions that drive DIV gene expression and further examinations of the mechanisms that control virus-mediated bloom control in diatoms. Moreover, the identified ClP1 promoter can serve as a novel tool for metabolic engineering of diatoms. This is the first report describing a promoter of DIVs that may be of use in basic and applied diatom research.

Diatoms are one of the most effective and diverse unicellular photosynthetic eukaryotes, with perhaps as many as 200,000 extant species found in aquatic environments^1^. There is broad interest in diatoms due to their substantial contributions to global carbon cycling and oxygen production^2^, their complex evolutionary background as secondary endosymbionts^3^, and their unique capacities to produce silica-based cell walls^4^. Moreover, diatoms present great potential as a source of beneficial chemicals for use in human activities because they produce biofuel precursors such as fatty acids and hydrocarbons that may prove useful in solving ecological problems such as the energy crisis^5^. To understand diatom biology and the means of diatom exploitation in biotechnology, novel genomic resources and molecular tools have been developed in recent years. Databases of genome sequences and the expressed sequence tag (EST) of the model diatoms Pennales *Phaeodactylum tricornutum* and Centrics *Thalassiosira pseudonana* have been released for public use, allowing the identification of distinct metabolic features in diatoms^6^,^7^,^8^,^9^. Recently, the genomic sequences of other diatoms such as *Pseudo-nitzschia multiseries* (http://genome.jgi-psf.org/Psemu1/Psemu1.home.html) and *Fragilariopsis cylindrus* (http://genome.jgi-psf.org/Fracy1/Fracy1.home.html) have also been made available on the U.S. Department of Energy Joint Genome Institute’s website (http://jgi.doe.gov/). In addition, methods of genetic transformation via biolistic bombardment and/or electroporation have been developed for several diatom species including Pennales *P. tricornutum*^10^,^11^,^12^,^13^,^14^,^15^,^16^, *Cylindrotheca fusiformis*^17^,^18^, *Navicula saprophila*^19^, *Pseudo-nitzschia arenysensis*^20^, *Pseudo-nitzschia multistriata*^20^, Centrics *Cyclotella cryptica*^19^, *Thalassiosira pseudonana*^21^, *Thalassiosira weissflogii*^22^, *Chaetoceros* sp.^23^, *Cha. gracilis*^24^, and *Fistulifera* sp.^25^. Moreover, episomal vector technology based on yeast-derived sequences has recently been developed for *P. tricornutum* and *Thalassiosira pseudonana*^26^.

However, many of the processes that control diatom biology and growth in marine environments remain to be elucidated. Recently, it has been proposed that marine viruses play a key role in controlling the blooms of diatom populations^27^,^28^. Viruses may also influence the compositions of marine communities and may act as a major force behind biogeochemical cycles^29^,^30^. Thus far, several marine diatom-infecting DNA/RNA viruses that cause the lysis of host diatoms have been isolated from both Centrics and Pennales diatoms^28^, for example, the *Cha. debilis*-infecting DNA virus (CdebDNAV)^31^, the *Cha. lorenzianus*-infecting DNA virus (ClorDNAV)^32^, and the *Thalassionema nitzschioides*-infecting DNA virus (TnitDNAV)^33^. *In silico* analysis of these genomes has revealed the presence of putative open reading frames (ORFs) such as the replication-associated protein (VP3) gene, the structural protein (VP2) gene, and genes of unknown function^28^,^32^.

To determine the contributions of marine diatom-infecting viruses (DIVs) to phytoplankton biology and ecology, it is important to further examine the DIV genome and to identify mechanisms that control viral gene expression through the characterization of viral promoters. Moreover, DIV promoter characterization may also advance the manipulation and modulation of gene expression in diatoms. Thus far, this strategy is used primarily through endogenous promoters such as the gene-encoding fucoxanthin chlorophyll *a*/*c*-binding protein (FCP) gene (*fcp,* now called *Lhcf*), which encodes members of the light harvesting complex superfamily^10^,^18^,^21^,^24^, frustulin^17^, nitrate reductase (NR)^18^,^21^,^24^, acetyl-CoA acetyltransferase^24^, acetyl-CoA carboxylase^19^, β-carbonic anhydrase 1 (CA1)^34^, long-chain fatty acyl-CoA synthetase^24^, β-tubulin^24^, ATP synthase subunit C^24^, and elongation factor 2 (EF2)^16^.

Of the endogenous promoters, the *fcp* promoter has been frequently used in biotechnological applications involving diatoms^35^,^36^,^37^,^38^,^39^. However, reports also suggest that *fcp* promoter activity may not be strong enough to overexpress introduced genes and may promote significant increases in target products^35^,^39^. Other studies report that transgene expression follows a circadian pattern due to the presence of light-responsive *cis*-regulatory elements in the *fcp* promoter^16^,^40^,^41^. In addition, in some cases, diatom endogenous gene promoters drive species-specific transgene expression and do not function in different diatom species^18^,^20^,^22^. These issues considerably limit the applications of diatom transformation techniques, potentially reducing the utility of diatoms in basic and applied research. To address these problems, the development of promoters that stably induce high-level gene expression is anticipated, which should result in the creation of large accumulations of introduced gene transcripts and proteins. Promoters from viruses that infect plants or mammals have generally been used to transform a wide range of higher plants and mammals, allowing the constitutive and strong expression of introduced genes. For example, the cauliflower mosaic virus 35S (CaMV 35S) and cytomegalovirus (CMV) promoters are known to efficiently facilitate plant and mammalian transformations, respectively^42^,^43^. However, only a few reports focus on the transformation of diatoms through the use of CaMV 35S and CMV promoters^19^,^44^ in *P. tricornutum,* and the same promoters do not seem to be effective in other species, such as the Centrics diatom *Cyc. cryptica*^19^.

DIV promoters may enable us to solve the above-described limitations in the bioengineering of diatoms as well as to infer the ecological roles of DIVs in marine ecosystems. Thus, in this study, potential promoter regions located upstream of ORFs, such as the replication-associated protein (VP3) gene and the structural protein (VP2) gene in the DIV genome (Fig. 1a), were isolated. DIV promoter-driving gene expression capacities in the Pennales diatom *P. tricornutum* were evaluated and compared to those of the endogenous *fcp* promoter (Fig. 1b). We then examined the stability of DIV promoters in cells that were cultured under various growth conditions. The effects of the growth stage, the nutrient concentrations in media, and the photoperiods on viral promoter activity were investigated. The application of viral promoters to a Centrics diatom and to unicellular green alga was also examined. We then discussed the structure of a DIV promoter to show how gene expression mechanisms may play a key ecological role in the control of diatom blooms and how the DIV promoter may be used in applied research on diatoms.

## Results

### Isolation of potential DIV promoter regions

**Figure 1:**
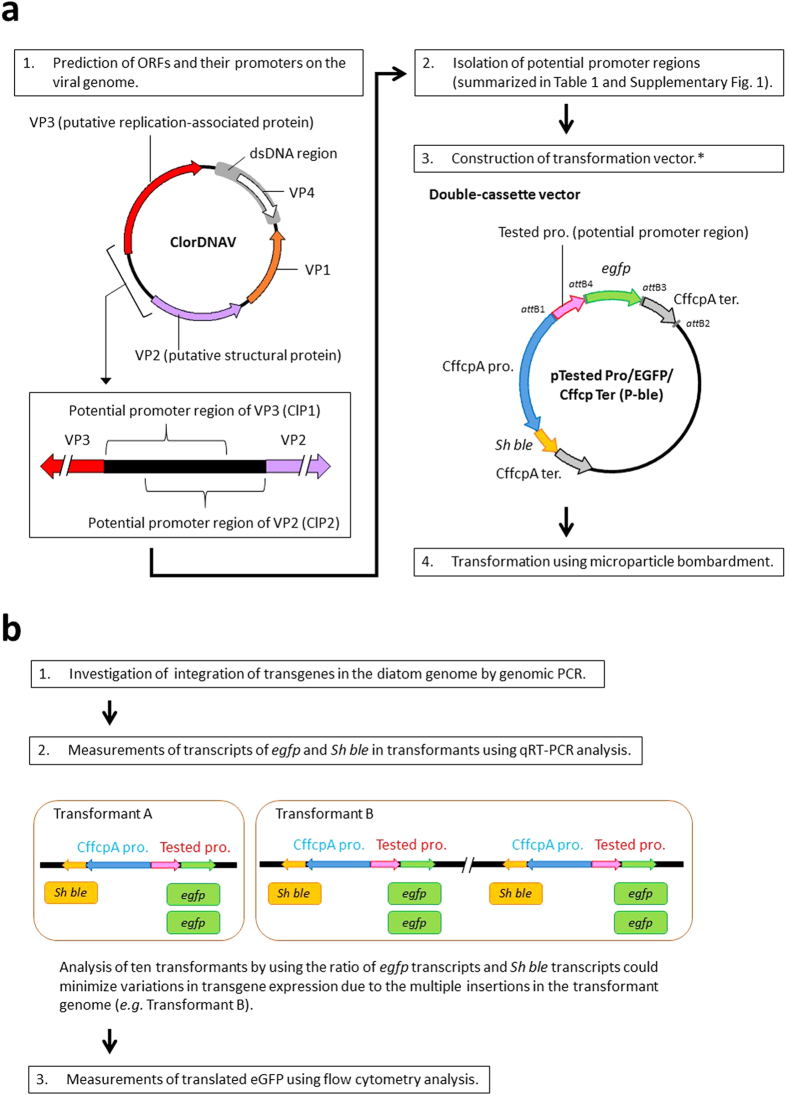
Schematic diagram for the evaluation of promoter activity. (**a**) Outline of the construction of transformation vectors and transformations. After predicting putative ORF positions^32^, upstream regions of the ORFs were determined as potential promoter regions. Potential promoter regions amplified by PCR were used to construct the transformation vectors. The double-cassette vector containing the reporter gene *egfp* driven by each tested promoter and the antibiotic-resistant gene *Sh ble* driven by the promoter region of the fucoxanthin chlorophyll *a*/*c*-binding protein (FCP) A-1A gene derived from *Cyl. fusiformis* (termed CffcpA pro.) were constructed. (**b**) Assessment of promoter activity. Promoter activity was determined by averaging the ratios of *egfp* mRNA transcript levels to those of *Sh ble* mRNA transcripts in ten transformants to minimize the effects of copy numbers on the expression of transgenes. These transformants were also used to investigate eGFP protein expression patterns. CffcpA ter.: terminator region of the FCP A-1A gene derived from *Cyl. fusiformis*. The structure of the ClorDNA genome was modified from Tomaru *et al.*^32^. *For the transformation vector of the nitrate reductase gene promoter, we used pNICgfp^18^ (Supplementary Fig. 5a).

Potential DIV promoter regions were determined from the genome sequences of CdebDNAV and TnitDNAV via *in silico* analyses. The ORFs of the two DIVs were identified using an ORF finder (National Center for Biotechnology Information, http://www.ncbi.nlm.nih.gov/gorf/). Three (VP1, VP2, and VP3) and two (VP2 and VP3) ORFs were identified in the CdebDNAV and TnitDNAV genomes, respectively. In regard to ClorDNAV, the four ORFs (VP1, VP2, VP3, and VP4) were previously described by Tomaru *et al.*^32^ (Fig. 1a). Regions (approximately 500 bases long) upstream of the translation initiation codon (ATG) of a putative ORF in the DIV genome were predicted to be potential promoter regions (Table 1, Fig. 1a, and Supplementary Fig. 1).

### Construction of transformation vectors

The potential promoter region of each ORF was amplified via polymerase chain reaction (PCR) using DIV genomic DNA and specific primers (Supplementary Table 1). In addition to the potential DIV promoters, the endogenous *fcp* promoter of *P. tricornutum* and some extrinsic promoters, such as CaMV 35S, CMV, and the nopaline synthase gene (*nos*) promoter of *Agrobacterium tumefaciens*, were amplified from the genomic DNA of *P. tricornutum* and from commercially available vectors, respectively, via PCR amplification (Supplementary Table 1). To investigate the activity of each tested promoter, we constructed a double-cassette transformation vector (Fig. 1a) that contained the enhanced green fluorescence protein (eGFP) gene (*egfp*) driven by each tested promoter and the antibiotic-resistant gene *Sh ble* driven by the *Cyl. fusiformis* FCP A-1A gene promoter (termed CffcpA pro.). We also constructed a single-cassette transformation vector containing *Sh ble* driven by each tested promoter (Supplementary Fig. 2) and *nat* driven by DIV promoters, such as CdP1 and ClP1 (Supplementary Fig. 3), to investigate transformation efficiency.

### Confirmation of the introduced gene and promoter in the transformants

Colonies were obtained from a solid f/2 medium containing 500 μg ml^−1^ antibiotics after its transformation via microparticle bombardment using the double-cassette transformation vectors. To confirm the introduction of the two cassettes, a genomic PCR analysis was performed using different primer sets (Supplementary Tables 1 and 2 and Supplementary Figs 4a and 5a) and using the colony-forming cells as templates. We obtained more than ten transformants with the expected DNA fragment length and containing *egfp* with the tested promoter, with the exception of transformants that were transfected with pNICgfp^18^. Six transformants that had been transfected with pNICgfp^18^ possessed DNA fragments of the expected length, including *egfp* under the control of the NR gene (*nr*) promoter from *Cyl. fusiformis* (termed Cfnr pro.) and the *Sh ble* gene under the CffcpA pro. A typical electrophoretogram of the amplicons in each transformant is shown in Supplementary Figs 4b and 5b. Ten transformants with each of the tested promoters and six transformants with Cfnr pro. were analysed further.

### Quantitative analysis of promoter activity

**Figure 2:**
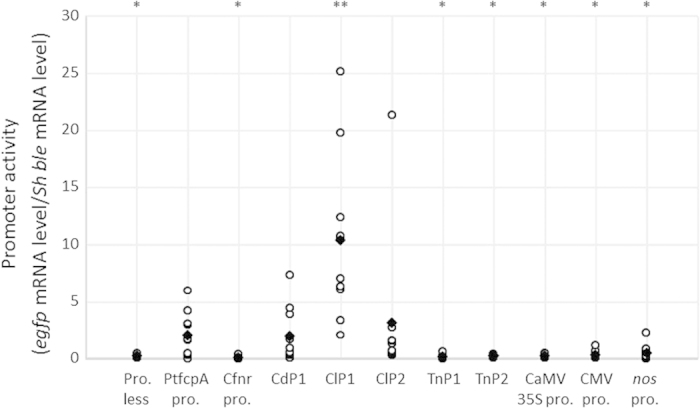
Relative activities of various promoters including DIV promoters in transformants. Ten independent transformants for each promoter were analysed. The promoter activity levels were determined by dividing *egfp* mRNA levels by *Sh ble* mRNA levels. The circles indicate the mean triplicate measurements of the independent transformants. The diamonds denote the average values of the six transformants from Cfnr pro. or of the ten transformants from the other promoters. Pro.-less denotes cases in which no promoter was linked to the *egfp* gene in the transformation vector (negative control). PtfcpA pro. shows the positive control in the transformation vector *egfp* gene driven by PtfcpA pro. Asterisks indicate the presence of a statistically significant difference derived from the PtfcpA pro. transformants (**P < 0.01 and *P < 0.05).

Variations in the levels of *egfp* mRNA transcripts normalized to the internal standard gene (ribosomal protein small subunit 30S gene, *rps*) mRNA transcripts were detected using quantitative reverse transcription-PCR (qRT-PCR) in 6−10 transformants with the *egfp* driven by each of the tested promoters (Supplementary Fig. 6). Variations in the levels of *Sh ble* mRNA transcripts driven by the diatom endogenous promoter (CffcpA pro.) that had been normalized to *rps* were also found amongst the 6−10 transformants (Supplementary Fig. 6). Before determining the activity of each promoter, the *egfp* mRNA transcript levels in each transformant were divided by those of the *Sh ble* mRNA transcripts. This method has been used to compare gene expression levels in independent lines and to prevent misinterpretation due to the presence of different transgene copy numbers and possible site integration effects. Figure 2 shows averages and ranges of promoter activity in the 6−10 independent transformants with *egfp* driven by each tested promoter. We compared the activity of each tested promoter with that of an endogenous promoter PtfcpA pro. because the PtfcpA pro. has generally been used for the transformation of the marine diatom *P. tricornutum*^10^,^11^,^13^,^39^. Average promoter activities in the transformants with Cfnr pro. (*P* = 0.010), TnP1 (*P* = 0.012), TnP2 (*P* = 0.016), the CaMV 35S promoter (*P* = 0.014), the CMV promoter (*P* = 0.021), and the *nos* promoter (*P* = 0.034) (average: 0.0833−0.499) were found to be significantly lower than those in the transformants with PtfcpA pro. (average: 2.08, range: 0.0296−5.97) (Fig. 2). By contrast, the average promoter activity in ClP1 (average: 10.3, range: 2.06−25.1, *P* = 0.0058) was found to be approximately five times higher than that in PtfcpA pro. (Fig. 2). The average CdP1 (average: 1.99, range: 0.0917−7.34, *P* = 0.93) and ClP2 activity (average: 3.09, range: 0.352−21.4, *P* = 0.64) levels showed no significant differences from the average PtfcpA pro. activity level (Fig. 2). The maximum activity levels in the ten transformants of CdP1, ClP1, and ClP2 were higher than that found in the transformants with PtfcpA pro. (Fig. 2).

### Effect of culture conditions on DIV promoter activity

**Figure 3:**
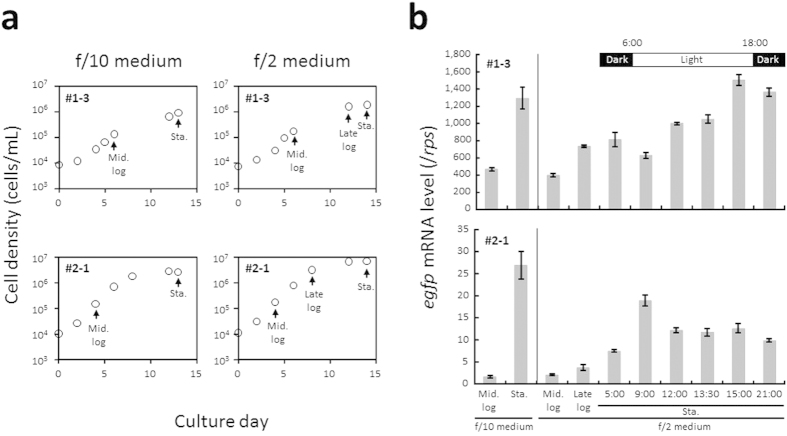
Analyses of viral promoter activities in different culture conditions. (**a**) Growth of two transformants with ClP1-driven *egfp* in f/10 medium and f/2 medium. (**b**) Relative abundances of *egfp* mRNA determined by dividing *egfp* mRNA transcript levels by those of ribosomal protein small subunit 30S gene (*rps*; internal control gene) mRNA transcripts in the transformant cells incubated in f/10 and f/2 media and harvested at various growth phases and at different times during a light/dark period. The arrows show cell collection points for the qRT-PCR analysis.

Promoter activities are known to respond to changes in environmental and intracellular conditions. To explore the effects of culture conditions and growth phases on the activity of the DIV promoter (ClP1) that showed the highest levels of activity (Fig. 2), the abundance of *egfp* mRNA levels in two independent ClP1 transformants was analysed using qRT-PCR under various culture conditions (Fig. 3). Under a low nutrient culture condition (f/10 medium), the abundance of *egfp* mRNA transcripts driven by ClP1 was almost identical to that reported for the standard nutrient culture condition (f/2 medium) (Fig. 3b). The abundance of *egfp* mRNA transcripts in the stationary phase seemed to be higher than that found for the log phase under both low and standard nutrient culture conditions (Fig. 3b). ClP1 can effectively drive the expression of *egfp* mRNA transcripts in both light and dark conditions during the stationary phase (Fig. 3b).

### Levels of eGFP fluorescence

**Figure 4:**
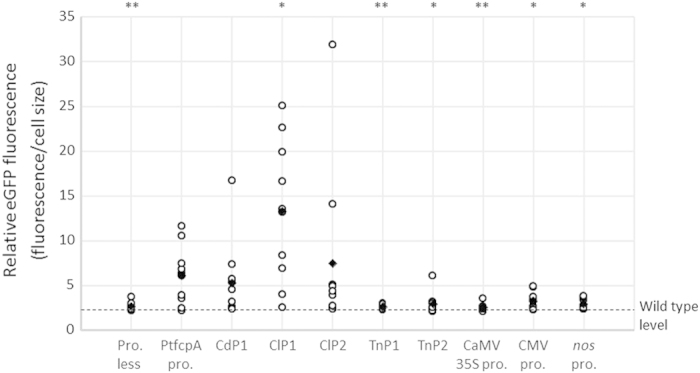
Relative abundance of eGFP protein translated from transcripts driven by various promoters in transformants using flow cytometry. Ten independent transformants derived from various promoters with the exception of the CaMV 35S promoter were analysed. For the CaMV 35S promoter, nine independent transformants were analysed. For normalization purposes, the eGFP fluorescence was divided by the cell size, which was estimated based on values of forward scatter areas (FSC-A) obtained via flow cytometry. The circles denote the mean value for approximately 10,000 cells of independent transformants. The diamonds denote the average values of the transformants. Asterisks indicate statistically significant differences derived from the PtfcpA pro. transformants (**P < 0.01 and *P < 0.05). The broken line denotes the autofluorescence level of wild type cells excited at 488 nm.

The fluorescence of the eGFP reporter in the different transgenic lines described above was quantified via flow cytometry. Figure 4 shows normalized eGFP fluorescence levels for cell sizes in the 9−10 independent transformants with *egfp* driven by each tested promoter. The average eGFP fluorescence levels in the transformants containing TnP1 (*P* = 0.0071), TnP2 (*P* = 0.012), the CaMV 35S promoter (*P* = 0.0063), the CMV promoter (*P* = 0.021), and the *nos* promoter (*P* = 0.012) (average: 2.53−3.24) were found to be significantly lower than those of the transformants containing PtfcpA pro. (average: 6.10, range: 2.21−11.6) (Fig. 4). The statistical analysis results show that the average eGFP fluorescence levels in the transformants containing ClP1 (average: 13.2, range: 2.52−25.0, *P* = 0.020) were significantly higher than that of the transformants containing PtfcpA pro. The average eGFP fluorescence levels in the transformants with CdP1 (average: 5.27, range: 2.38−16.7, *P* =0.63) and ClP2 (average: 7.45, range: 2.37−31.8, *P* = 0.67) showed no significant differences from that in the transformants with PtfcpA pro. (Fig. 4). The maximum levels of eGFP fluorescence in the transformants of CdP1, ClP1, and ClP2 were found to be higher than that in the transformants of PtfcpA pro. (Fig. 4). The eGFP fluorescence levels without normalization were similar to those observed by normalizing fluorescent signals with cell sizes (Supplementary Fig. 7).

### Transformation efficiency using DIV promoters

We also assessed whether the different promoters identified in this study may affect transformation efficiency levels in *P. tricornutum*. To this end, single-cassette vectors containing the selective marker, *Sh ble*, driven by PtfcpA pro. or ClP1 (Supplementary Fig. 2), were introduced into *P. tricornutum* via microparticle bombardment. The average transformation efficiencies of PtfcpA pro. and ClP1 reached 13 and 13.5 transformants per 10^8^ cells (n = 4), respectively. Statistical analysis based on the number of colonies formed showed that the transformation efficiency using PtfcpA pro. and ClP1 were not significantly different.

### Application of diatom-infecting DIV promoters to other algae

We investigated whether the DIV promoter may also be applied to a species of Centrics diatoms such as *Chaetoceros* sp. Strain CCK09. To this end, we assayed transformation efficiency levels in this species using single-cassette vectors with the antibiotic-resistant gene *nat* driven by the DIV promoters CdP1 and ClP1 (Supplementary Fig. 3). The average transformation efficiencies of CdP1 and ClP1 transfected to *Chaetoceros* sp. were 4 and 2 transformants per 10^8^ cells (n = 3), respectively.

The activity levels of the DIV promoters in the unicellular green alga *Chlamydomonas reinhardtii*, a species that is phylogenetically different from the diatoms, were also investigated. Single-cassette vectors, in which *She ble* was driven by a DIV promoter (for example, CdP1 and ClP1) or the *Chlamydomonas* endogenous promoter *rbcS2* (pSP108)^45^, were used for the assay. A standard electroporation method^46^ and wall-less *Chlamydomonas* strain CC503 were used for the transformation. The transformation efficiency of pSP108 was recorded at 168 transformants per 2.5 × 10^7^ cells (n = 14). By contrast, the average transformation efficiency found via CdP1 and ClP1 were recorded at 0 and 0.14 transformants per 2.5 × 10^7^ cells (n = 14), respectively. The transformation efficiency levels obtained using viral promoters were not significantly different from those of the promoter-less vector (average: 0 transformant, n = 14).

### *In silico* analyses of DIV promoter regions

We searched for potential *cis*-regulatory elements in the DIV promoters using the PLACE^47^ and PlantCARE^48^ programs. Both programs revealed the existence of *cis*-regulatory elements of Myb^49^, bZIP^50^, CCAAT-binding^51^, homeobox^52^, and E2F-DP^53^-related transcription factors (TFs) (which have already been found in genomic sequences of the Pennales *P. tricornutum* and the Centrics *Thalassiosira pseudonana*^54^) in the DIV promoters (Supplementary Fig. 1). The PlantCARE program^48^ also showed that all the DIV promoter regions include a plant-type light-responsive *cis*-regulatory element (Supplementary Fig. 1). To identify other regulatory sequences that are conserved amongst DIV and endogenous promoters, we examined the frequency of consensus TF binding sites (TFBSs), which are available in the JASPAR database^55^, through promoterome analyses^41^ using oPOSSUM version 3^56^. The single site analysis (SSA) algorithm of oPOSSUM version 3 failed to detect any TFBS consensus between the DIV promoters and the potential promoter regions of *P. tricornutum*. Using the TFBS cluster analysis (TCA) algorithm available in oPOSSUM version 3, sequence motifs of some insect- and vertebrate-type homeobox binding sites were identified in the five DIV promoters and in the 99.9% potential promoter region of *P. tricornutum* (12,234 of 12,237 sequences, Z-score: 14.406, Fisher score: 0.001) (Supplementary Fig. 1 and Supplementary data). To obtain information on specific mechanisms that drive expression from DIV promoters, we attempted to find conserved motifs among the DIV promoter regions (CdP1, ClP1, and ClP2) using a consensus motif-finding algorithm (CONSENSUS) available through Melina II^57^ and using default parameters. The “GGCAGGCG” sequence was detected in CdP1, ClP1, and ClP2 through the CONSENSUS algorithm. In the sense strand of ClP1, there are three tandem repeats of the “GGCAGGCG” motif ordered from 5′ to 3′ (Supplementary Fig. 1). In ClP2, there are three tandem repeats, with the direction of the motifs ordered from 3′ to 5′. Only one motif of the 5′ to 3′ direction was found in the sense strand of CdP1 (Supplementary Fig. 1).

## Discussion

This is the first report describing the functional characterization of various promoters identified and isolated from DIVs. We tested viral promoter activity levels in diatom cells transformed with specific vectors, in which the viral promoter drove the expression of an eGFP reporter gene. The activity of each tested promoter was estimated from the relative abundance of the eGFP gene transcript relative to that of the antibiotic-resistant gene driven by the endogenous *fcp* promoter (Fig. 1b). In turn, we identified the strongest promoters and overcame issues of gene expression variability between independent transgenic lines that can be due to differences in the copy numbers of transgenes integrated in a host genome^58^ and from the positioning of the introduced vector in the genome, i.e., the position effect^59^. Similar variations have been reported during the characterization of several promoters^60^. Moreover, variations in fluorescence levels of the eGFP protein were used to assess possible variations in translational efficiency levels in the different transgenic lines. These analyses allowed us to show for the first time that the isolated DIV promoters are functional in *P. tricornutum*. In particular, we found that the ClP1 promoter of a putative replication-associated (VP3) gene derived from ClorDNAV^32^ drives the stable expression of the reporter in cells grown in both light and dark conditions, and it shows higher levels of activity than a diatom endogenous promoter.

Interestingly, the activity levels of viral promoters may be heavily modulated in the growth phase. In *Schizosaccharomyces pombe*, gene expression mediated by the CaMV 35S promoter is induced during the log phase rather than during the stationary phase^61^. In our study, reporter expression controlled by ClP1 was induced at the stationary phase rather than at the log phase of *P. tricornutum* growth. In accordance with our data, a study on the relationship between the *Cha. tenuissimus*-infecting DNA virus (CtenDNAV) type II and the growth phase of *Cha. tenuissimus* showed that when CtenDNAV type II is inoculated into host cells at the stationary phase, a decrease in host cell quantities occurred one day after inoculation^28^. However, in host cells at the log phase, a decrease in host cell quantities occurred only after the cells reached the stationary phase^28^. The infectivity of CtenDNAV type I was also considerable when the host was at a stationary phase^62^. Moreover, an active replication of the viral genome of CtenDNAV type I was observed after the host cells reached the stationary phase^62^. Taken together, these results suggest that DIV promoter activation in a host diatom at the stationary phase may facilitate diatom lysis. Although gene expression regulation by DIV promoters in *P. tricornutum* is not fully understood, these findings suggest that a stronger understanding of the mechanisms that control DIV gene expression may lead to the development of a stronger understanding of diatom population control processes by DIVs in oceans.

Our assessment of viral promoter activity also allowed us to identify ClP1 as a novel DIV promoter that drives strong and stable gene expression in diatoms. ClP1 serves as a useful tool that may be exploited in diatom biotechnology applications for the high-level production of useful materials and for gene functional studies in genetically engineered diatoms^63^,^64^. The activity of the “stable” endogenous EF2 gene promoter recently isolated from *P. tricornutum*^16^ drove the expression of a transgene 1.2-fold stronger than that driven by the *fcp* promoter in light conditions^16^. In comparison, *egfp* mRNA levels normalized to *rps* in ClP1 transformants were found to be 3.3 times stronger than those of PtfcpA pro. transformants (Supplementary Fig. 6). This result suggests that ClP1 may induce a stronger expression of transgenes than the EF2 gene promoter throughout a photoperiod. In addition, ClP1 activity remains stable in low nutrient culture conditions. A combination of stable ClP1 activity levels in light/dark cycles under low nutrient conditions and high-level ClP1 activity levels in the stationary phase may have value in biotechnological applications.

In discussing whether DIV promoters may be applied to various diatom species, it is important to consider the phylogeny of diatoms composed of the two orders Pennales and Centrics^1^,^65^. In this study, the finding that ClP1 can be applied to both the Centrics diatom *Chaetoceros* sp. and the Pennales diatom *P. tricornutum* suggests that the DIV promoter region contains conserved regulatory elements that may be recognized via conserved TFs identified in Pennales and Centrics species^54^. Considering the phylogenetic relationship, conservation levels in TF families between the Pennales and Centrics diatoms, and our results, DIV promoters including ClP1 may be applied to various diatom species.

Both DIV promoters CdP1 and ClP1 are derived from *Chaetoceros* spp., although the transformation efficiency of *P. tricornutum* was found to be higher than that of *Chaetoceros* sp. It was reported that transformation efficiencies obtained by multi-pulse electroporation using the endogenous *fcp* promoter in *P. tricournutum*^13^ were approximately 10 times higher than those in *Chaetoceros gracilis*^24^, suggesting that the transformation efficiency levels in *Chaetoceros* are lower than those of *Phaeodactylum,* independent of the promoter used to drive the transgene expression. By contrast, ClP1 cannot function in green algae *Chl. reinhardtii*, suggesting that regulatory elements in promoter regions and transcriptional regulators differ between the Heterokont and Plantae lineages.

Via an *in silico* analysis of the DIV promoters, we examined conserved motifs that may be involved in the initiation of diatom virus gene expression. Topically, eukaryotic promoters consist of two regions: a core region and an upstream regulatory region^66^. The core region consists of an approximately 50−100 base pair (bp) sequence. It includes the transcription start site, known as an initiator (Inr), and flanking sequences such as the TATA box^67^. The regulatory region includes *cis*-regulatory elements that control gene expression through interaction with TFs^66^. *Cis*-regulatory elements mediate inducible, tissue specific, or development stage-specific gene expression^66^. In terms of Inr sequences, an Inr consensus has been found for some eukayotes^67^,^68^,^69^. Based on the transcription start site of *P. tricornutum*, the conserved sequence flanking the transcription start sites (CAY_+1_A, degenerate bases are described according to the IUPAC nucleotide code) is reported from the upstream regions of some FCP genes^70^. In addition, −2 to +2 sequences relative to the transcription start site of the CA1 gene (*ca1*) in *P. tricornutum* were found to be CACA^34^. We focused on sequences surrounding CAYA located in the upstream DNA sequence of FCP genes, and other endogenous genes of diatoms were examined via visual observation. We found a “TCAH_1_W” Inr-like sequence located between bp 26−61 upstream of the translation site in *P. tricornutum* FCP genes such as the *fcpC*, *fcpD* and *fcpE*, and *P. tricornutum* CA1 genes (Supplementary Table 3). In the tested DIV promoter regions, we also found this Inr-like sequence between bp 14−65 upstream of the proposed translation site (CdP1: 14 and 35 upstream, ClP1: 36 and 61 upstream, ClP2: 40 upstream, TnP1: 35 upstream, and TnP2: 65 upstream) (Supplementary Table 3 and Supplementary Fig. 1). To determine the universality of “TCAH_+1_W” in the 5′-flanking sequences (80 bases) of the *P. tricornutum* genes, we searched for “TCAH_+1_W” in 12,237 of the 5′-flanking sequences and found “TCAH_+1_W” in 8,306 of the 12,237 sequences (67.9%) of the potential promoter region of the *P. tricornutum* genes. From these findings, we present a new consensus motif “TCAH_+1_W”, which serves as a potential Inr-like sequence in the diatom.

While identifying other regulatory sequences that are conserved amongst DIV and endogenous promoters, we found sequence motifs of some insect- and vertebrate-type homeobox binding sites in the five DIV promoters and in the 99.9% potential promoter region of *P. tricornutum* via a promoterome analysis^41^. This finding suggests that these motifs may play a role in *P. tricornutum* transcription.

In addition to revealing the existence of canonical *cis*-regulatory elements, several studies have reported on the existence of other *cis*-regulatory elements in diatom endogenous gene promoters. Three putative *cis*-regulatory elements from the *ca1* promoter of *P. tricornutum,* which responds to changes in CO_2_ and cAMP concentrations, have been found^34^,^71^. The two iron-responsive conserved *cis*-regulatory elements were found in upstream DNA sequences of iron starvation-induced protein genes in *P. tricornutum*^72^. The expression levels of FCP genes were found to oscillate in a circadian manner in response to a plant-type light-responsive *cis*-regulatory element in the *fcp* promoter^40^,^41^,^73^,^74^,^75^. *Cis*-regulatory elements of CO_2_/cAMP^71^ or iron^72^ were not found in any of the DIV promoter regions by visual observation. The PlantCARE program^48^ showed that all the DIV promoter regions possess a plant-type light-responsive *cis*-regulatory element. However, ClP1 activity was unaffected by light and dark cycles (Fig. 3), suggesting that the identified elements do not respond to light. EF2 gene promoter activity was also found to be stable in *P. tricornutum* throughout light and dark cycles. In the sequence of the promoter region of the EF2 gene, a plant-type light-responsive *cis*-regulatory element was also detected through the PlantCARE program^48^, supporting the notion that plant-type light-responsive *cis*-regulatory elements cannot respond to light in *P. tricornutum*.

The “GGCAGGCG” sequence was detected in CdP1, ClP1, and ClP2 via the CONSENSUS algorithm. The quantity, direction, and proximity of this consensus motif varied among these promoters. ClP1, which showed the highest degree of promoter activity (Fig. 2), possessed three tandem repeats of the “GGCAGGCG” motif in the sense strand in the 5′-to-3′ direction. For ClP2, which showed modest activity levels (Fig. 2), a 3′-to-5′ direction was found, even when three tandem repeats were present in the promoter region (Supplementary Fig. 1). Only one motif was found in the sense strand of CdP1, which also showed modest activity levels (Fig. 2 and Supplementary Fig. 1). However, one and three “GGCAGGCG” sequences were found in the antisense strands of the *nos* and CMV promoters, respectively, which showed their very weak activity levels (Fig. 2). In the latter case, the three motifs are scattered across the promoter region (Supplementary Fig. 8). These findings suggest that the quantity, direction, and proximity of the novel consensus motif may heavily affect the stability of promoter activity levels though light/dark cycles and of high-level promoter activity levels during the stationary phase in *P. tricornutum*. In addition to the motifs described above, unknown novel motifs may exist in the promoter regions of DIVs such as ClP1.

In summary, we have isolated promoters from several marine DIVs for the first time. The activity of the DIV promoter of ClP1 in the transformants was higher in the stationary phase than it was in the log phase. Compared to a diatom endogenous promoter, the mediated ClP1 showed a significantly higher level of reporter transcription and translation. Conservative motifs in sequences of the DIV promoters were found. Finally, our findings may help elucidate the mechanism of DIV gene expression, which may play a key role in the formation/decay of diatom blooms in oceans. In addition, the stable and high ClP1 promoter activity levels reported should prove useful for the metabolic engineering of diatoms.

## Methods

### Algal culture

The Pennales *P. tricornutum* Bohlin (National Research Institute of Aquaculture, Fisheries Research Agency, Japan, strain NRIA-0065), the Centrics *Chaetoceros* sp. (Japan Marine Science and Technology Center, Japan, strain CCK09)^23^, and unicellular green alga *Chl. reinhardtii* (strain CC-503) were used in this study. *P. tricornutum* was grown at 20 °C under a light/dark cycle of 12 h light and 12 h dark with *ca*. 90 μmol photons m^−2^ s^−1^ in liquid f/2 medium^76^. *Chaetoceros* sp. was grown at 20 °C under continuous illumination at 300 μmol photons m^−2^ s^−1^ in SWM3 medium^77^. The *Chl. reinhardtii* strain CC-503 was provided by the *Chlamydomonas* Resource Center (University of Minnesota). The cells were cultivated mixotrophically at 25 °C in Tris-acetate phosphate (TAP) medium^78^ under constant white fluorescent light conditions (84 μmol photons m^−2^ s^−1^) with gentle shaking.

### Isolation of DIV promoter regions

DIVs and the sequences of the viral genome were isolated following the method described by Tomaru *et al.*^32^. In brief, a 450 ml exponentially growing diatom culture was inoculated with 5 ml of DIV suspension and then lysed. The lysate was filtrated through a 0.4 μm pore size polycarbonate membrane filter (Whatman^®^; GE Healthcare UK Ltd., Buckinghamshire, England) for the removal of cellular debris. The filtrated lysate was mixed with polyethylene glycol 6000 (final concentration: 10% wt/vol; Wako Pure Chemical Industries, Ltd., Osaka, Japan), and the mixture was stored at 4 °C in the dark overnight. To collect the virus particles, after centrifugation at 57,000 × *g* at 4 °C for 1.5 h, the pellet was washed with 10 mM phosphate buffer (pH 7.2) and centrifuged at 217,000 × *g* and 4 °C for 4 h. Viral genomic DNA was extracted from the pellet using a DNeasy Plant Mini Kit (QIAGEN Inc., CA) according to the manufacturer’s instructions. We isolated three viral genomes: the CdebDNAV^31^ genome, the ClorDNAV^32^ genome, and the TnitDNAV^33^ genome. ORFs of ClorDNAV^32^ have been identified by Tomaru *et al.*^32^. We identified ORFs of CdebDNAV^31^ and TnitDNAV^33^ using an ORF finder following the method reported by Tomaru *et al.*^32^. Fragments of upstream regions of the ORFs, such as the replication-associated protein (VP3) gene and the structural protein (VP2) gene as promoter regions (Fig. 1a), were amplified using PCR. The isolated viral promoters and the source of the promoters are summarized in Table 1. The primers used in this study are shown in Supplementary Table 1. All the DNA fragments, including those in the promoter regions, were amplified using PrimeSTAR^®^ GXL DNA Polymerase (Takara Bio Inc., Otsu, Japan) according to the manufacturer’s instructions.

### Amplification of DNA fragments for vector construction

To construct transformation vectors (Fig. 1 and Supplementary Figs 2 and 3), DNA fragments containing various promoter regions, a reporter gene (*egfp*), antibiotic-resistant genes such as the bleomycin-resistant gene (*Sh ble*) and the nourseothricin-resistant gene (*nat*), and a terminator region were cloned into Gateway^®^ Pro Donor vectors using a Gateway^®^ cloning system according to the instructions of MultiSite Gateway^®^ Pro (Life Technologies Corporation, CA) for the preparation of entry vectors containing each fragment. DNA fragments containing PtfcpA pro., a CaMV 35S promoter, a CMV promoter, and a *nos* promoter were amplified from the genomic DNA of the *P. tricornutum* UTEX 646 strain (The Culture Collection of Algae (UTEX, The University of Texas at Austin)), pCAMBIA2301 (CAMBIA: Center for Application of Molecular Biology for International Agriculture, Canberra, Australia), phMGFP (Promega, WI), and pBI101.2 (Clontech, CA); these were used as templates. The *egfp* fragment was amplified from pNICgfp^18^ as a template. The terminator region of the FCP A-1A gene derived from *Cyl. fusiformis* (termed CffcpA ter.) was also amplified from pNICgfp^18^ as a template. The terminator region of the FCP gene derived from *Thalassiosira pseudonana* (termed Tpfcp ter.) was amplified from pTpfcp/nat^21^ as a template. Antibiotic genes, *Sh ble*, and *nat* were amplified from pNICgfp^18^ and pTpfcp/nat^21^ as templates. All the DNA fragments were amplified using PrimeSTAR^®^ GXL DNA Polymerase (Takara Bio Inc., Otsu, Japan), with the primer added to the *att* sites in the Gateway^®^ system (Supplementary Table 1) according to the manufacturer’s instructions. The amplicons were purified via agarose gel electrophoresis using a QIAquick^®^ Gel Extraction Kit (QIAGEN Inc., CA).

### Construction of double-cassette vectors

To evaluate promoter activity levels based on different transgene copy quantities and possible site integration effects, we prepared double-cassette vectors as shown in Fig. 1 using the Gateway^®^ cloning system according to the instructions for MultiSite Gateway^®^ Pro (Life Technologies Corporation, CA). To construct entry vectors, DNA fragments with various promoter regions, a reporter gene (*egfp*), and CffcpA ter. were cloned into pDONR221P1-P4, pDONR221P4r-P3r, and pDONR221P3-P2, respectively, using the Gateway^®^ BP Clonase^®^ II enzyme mix (Life Technologies Corporation, CA) according to the instructions designated for MultiSite Gateway^®^ Pro (Life Technologies Corporation, CA). In cases involving the construction of the promoter-less vector, the *egfp* gene and CffcpA ter. were cloned into pDONR221P1-P5r and pDONR221P5-P2, respectively. To construct double-cassette vectors, a destination vector was prepared by cloning the Gateway^®^ cloning cassette and Reading Frame Cassette A (Life Technologies Corporation, CA) into the *Kpn*I site of pCfcp-*ble*^18^ that contained the antibiotic gene *Sh ble* driven by CffcpA pro. The LR recombination reactions, which allow the recombination of three (promoter-reporter gene-terminator) or two fragments (reporter gene-terminator) from the entry vector into the destination vector, were conducted using the Gateway^®^ LR Clonase^®^ II PLUS enzyme mix (Life Technologies Corporation, CA) according to the instructions designated for MultiSite Gateway^®^ Pro (Life Technologies Corporation, CA). Finally, we constructed the transformation vector described as pTested Pro/EGFP/Cffcp Ter (P-ble) (Fig. 1a). To test the activity of the promoter from Cfnr pro., we used pNICgfp^18^ (Supplementary Fig. 5a).

### Construction of single-cassette vectors

To determine transformation efficiency, the single-cassette vectors shown in Supplementary Figs 2 and 3 were constructed following the method for the construction of double-cassette vectors. The antibiotic genes (*Sh ble* and *nat*) and terminator regions (CffcpA ter. And Tpfcp ter.) were cloned into pDONR221P4r-P3r and pDONR221P3-P2, respectively, according to the instructions for MultiSite Gateway^®^ Pro (Life Technologies Corporation, CA). To construct the promoter-less vector, the antibiotic gene and terminator were cloned into pDONR221P1-P5r and pDONR221P5-P2, respectively. To construct single-cassette vectors, the destination vector was prepared by cloning the Gateway^®^ cloning cassette, Reading Frame Cassette A (Life Technologies Corporation, CA), into the *Eco*RI site of the pBluescript SK(-) (Agilent Technologies, CA). The LR recombination reactions, which allow the recombination of three fragments (promoter-antibiotic gene-terminator) or two fragments (antibiotic gene-terminator) from the entry vector into the destination vector, were performed using the Gateway LR Clonase II PLUS enzyme mix (Life Technologies Corporation, CA) according to the instructions for MultiSite Gateway^®^ Pro (Life Technologies Corporation, CA). Finally, we constructed the single-cassette vectors (Supplementary Figs 2 and 3). For the transformation of *P. tricornutum*, PtfcpA pro. and ClP1 were examined. For the transformation of *Chaetoceros* sp. and *Chl. reinhardtii* CC-503, CdP1 and ClP1 were examined.

### Transformation of *P. tricornutum*

Transformation vectors were introduced into *P. tricornutum* cells using the Biolistic PDS-1000/He particle delivery system (Bio-Rad Laboratories, CA) according to a method previously described^10^,^12^,^23^. Approximately 1 × 10^8^ cells were spread on central areas covering one-third of a plate of solid f/2 medium containing 1% agarose HGS (Nacalai Tesque, Kyoto, Japan) *ca.* 1 h prior to bombardment. The plate was positioned on the second level (6 cm from the stopping screen) within the Biolistic chamber for bombardment. Tungsten particles M17 (3 mg, 1.1 μm diameter, Bio-Rad Laboratories, CA) were coated with 5 μg of the transformation vector in the presence of CaCl_2_ and spermidine for five shots, as instructed by the manufacturer. The cells were then bombarded with 600 ng of DNA-coated tungsten particles using a two-ply 650 psi (1300psi) rupture disk. After bombardment, the cells were recovered from the plates and incubated in liquid f/2 medium under standard growth conditions for 24 h. Cultures of *P. tricornutum* were concentrated by centrifugation and resuspended in 100 μl of f/2 medium. The cell suspensions were spread onto plates of solid f/2 medium containing 0.5% agarose HGS (Nacalai Tesque, Kyoto, Japan) supplemented with 500 μg ml^−1^ Zeocin^TM^ (InvivoGen, CA). After 5 weeks of selective incubation under standard growth conditions, individual colonies that had formed on the plates were extracted using a platinum loop and suspended into liquid media with 300 μg ml^−1^ Zeocin^TM^.

### Transformation of *Chaetoceros* sp

Single-cassette vectors for the transformation of Centrics diatoms (Supplementary Fig. 3) were introduced into *Chaetoceros* sp. cells using the Biolistic PDS-1000/He particle delivery system (Bio-Rad Laboratories, CA) according to a method previously described^23^. The cells were bombarded using a two-ply 650 psi (1300psi) rupture disk.

### Transformation of *Chl. reinhardtii* CC-50**3**

Nuclear transformation was performed using the electroporation method^46^. In brief, the cells were grown to 4 × 10^6^ cells ml^−1^ in TAP medium. Subsequently, 2.5 × 10^7^ cells were harvested by centrifugation and suspended in 250 μl of TAP medium supplemented with 50 mM sucrose (TAP/sucrose). Electroporation was performed by applying an exponential electric pulse of 0.7 kV at a capacitance level of 50 μF (Electro Cell Manipulator^®^ 600; BTX^®^ Harvard Apparatus, MA) and using 300 ng of non-linearized plasmids according to the manufacturer’s instructions. Transgenic strains were selected directly from TAP/agar plates containing Zeocin^TM^ (10 μg ml^−1^), and the plates were incubated under continuous fluorescent light conditions (20 μmol m^−2^ s^−1^) at 25 °C.

### PCR analysis of the introduced genes and of their promoters in the transformed cells

To determine whether the *egfp* gene with the tested promoters and the *Sh ble* gene with CffcpA pro. were introduced into colony-forming cells that showed resistance to Zeocin^TM^, regions containing the promoters and genes were amplified using the cells as a template. Genomic PCR analyses were conducted using Tks Gflex^TM^ DNA Polymerase (Takara Bio Inc., Otsu, Japan). The amplicons were analysed using agarose gel electrophoresis. The primers used in the genomic PCR analysis are listed in Supplementary Tables 1 and 2.

### RNA isolation

Total RNA was isolated from approximately 5 × 10^7^ transformed cells using an RNeasy Plant Mini Kit (QIAGEN Inc., CA) coupled with a RNase-Free DNase Set (QIAGEN Inc., CA). Reverse transcription (RT) was performed using a PrimeScript^®^ RT reagent Kit (Perfect Real Time) (Takara Bio, Otsu, Japan). We also used a SuperPrep^TM^ Cell Lysis & RT Kit for qPCR (TOYOBO, Osaka, Japan) to isolate total RNA and RT from some of the transformed cells.

### Assessment of relative promoter activities

qRT-PCR experiments to quantify *egfp* and *Sh ble* transcripts in the transformed cells were performed in triplicate on a Thermal Cycler Dice^®^ Real Time System Single MRQ (Takara Bio Inc., Otsu, Japan) using 2 μl of the cDNA mixture added as a template and SYBR^®^ *Premix Ex Taq*^TM^ II (Tli RNaseH Plus) (Takara Bio Inc., Otsu, Japan). The cycling conditions involved denaturing for 30 s at 95 °C and 40 cycles of melting (5 s at 95 °C) and annealing coupled with an extension (30 s at 60 °C). To determine promoter activities, each qRT-PCR measurement was performed in triplicate using primers to identify the abundance of *Sh ble* mRNA and *egfp* mRNA (shown in Supplementary Table 1). The *egfp* mRNA levels were normalized by dividing by the *Sh ble* mRNA levels to minimize variations in transgene expression due to multiple insertions (Fig. 1b).

### Effect of culture conditions on DIV promoter activity

To explore the effects of nutrient concentrations in media, growth phases of the tested strain, and light cycles on DIV promoter activity, two transformants with ClP1 were cultured in a low nutrient medium (f/10 medium) and standard nutrient medium (f/2 medium). Transformants at the log and stationary growth phases were collected at approximately 13:30. Transformants cultured in f/2 medium at the stationary growth phase were collected during various photoperiods. Total RNA samples from approximately 5 × 10^7^ transformed cells were isolated using the above methods. Using qRT-PCR, we determined the abundance of *egfp* mRNA and that of *rps* mRNA. The transcript number of *egfp* was divided by that of *rps*, the expression of which was found to be constitutive under various growth conditions^74^ and which was used as an internal control. The primers for detecting *rps* mRNA are listed in Supplementary Table 1.

### Flow cytometry

To measure the eGFP fluorescence for the transformed cells, approximately 1 × 10^4^ cells were analysed using a BD LSRFortessa™ X-20 flow cytometer system equipped with BD FACS Diva™ software (BD Biosciences, CA). The eGFP fluorescence was analysed using a 488 nm laser and a 530/30 nm bandpass filter. Endogenous chlorophyll fluorescence was analysed using a 488 nm laser and a 610/20 nm bandpass filter. We evaluated cell sizes by measuring the mean forward scatter area (FSC-A) using a 488 nm laser and a 488/10 nm bandpass filter. The data were analysed using FlowJo software (FlowJo, LLC, OR). The cells were gated on endogenous chlorophyll fluorescence so that debris could be eliminated from the analysis conditions. The average values of approximately 1 × 10^4^ cells for each sample were used for the statistical analysis.

### Statistical analyses

Statistical analyses were performed on the activity levels of the promoters at the transcriptional and translational levels using Student’s *t*-test for two group mean values (the PtfcpA pro. transformants and the other promoter transformants), and statistical significance was achieved when *P* < 0.01 (**) and *P* < 0.05 (*). In regard to the transformation efficiency of *P. tricornutum*, statistical analyses were performed using Student’s *t*-test for two group mean values (the colony number of PtfcpA pro. and that of ClP1). With regard to transformation efficiency in *Chl. reinhardtii*, statistical analyses were performed using Student’s *t*-test for two group mean values (the colony number of pSP108 and those of DIV promoters, or that of the promoter-less vector and those of DIV promoters).

### *In silico* analyses

Putative *cis*-regulatory elements in potential DIV promoter regions and other extrinsic promoters (the CaMV 35 S, CMV, and *nos* promoters) were identified by searching the PLACE^47^ and PlantCARE^48^ databases. The promoterome analysis was performed following the method described by Russo *et al.*^41^. In brief, we obtained 12,237 of the 5′-flanking sequences of the *P. tricornutum* genes from Ensembl Protists BioMart (Dataset: ASM15095v2 (2013-07-EBI-Phatr3))^79^,^80^. Using the oPOSSUM version 3^56^ program, SSA and TCA tests were performed using default parameters. The TFBS profile matrix file for vertebrates, insects, nematodes, and fungi was used in the SSA. For the TCA, the TFBS profile matrix file for vertebrates, insects, and nematodes was used. A consensus sequence for the amplified regions of the tested promoters was also analysed using the consensus motif-finding algorithms CONSENSUS from Melina II^57^ and using default parameters amongst potential DIV and extrinsic promoters (CaMV 35S, CMV, and *nos* promoters).

## Additional Information

**How to cite this article**: Kadono, T. *et al.* Characterization of marine diatom-infecting virus promoters in the model diatom *Phaeodactylum tricornutum*. *Sci. Rep.* **5**, 18708; doi: 10.1038/srep18708 (2015).

## Supplementary Material

Supplementary Information

Supplementary Dataset

## Figures and Tables

**Table 1:** Promoters used in this study.

Promoter	Source organism/virus	Promoter associated gene	Amplifiedsize (bp)	Ref.
CdP1	*Chaetoceros debilis*-infecting DNA virus (CdebDNAV)	Putative replication-associated protein (VP3) gene	477	31
ClP1	*Chaetoceros lorenzianus*-infecting DNA virus (ClorDNAV)	Putative replication-associated protein (VP3) gene	502	32
ClP2	*Chaetoceros lorenzianus*-infecting DNA virus (ClorDNAV)	Putative structural protein (VP2) gene	474	32
TnP1	*Thalassionema nitzschioides*-infecting DNA virus (TnitDNAV)	Putative replication-associated protein (VP3) gene	424	33
TnP2	*Thalassionema nitzschioides*-infecting DNA virus (TnitDNAV)	Putative structural protein (VP2) gene	424	33
PtfcpA pro.	*Phaeodactylum tricornutum*	Fucoxanthin chlorophyll *a/c*-binding protein A gene	444	10
Cfnr pro.	*Cylindrotheca fusiformis*	Nitrate reductase gene	774	18
CaMV 35S pro.	Cauliflower mosaic virus	35S gene	454	44,81
CMV pro.	Cytomegalovirus	Immediate early promoter regulatory gene	742	44,82
*nos* pro.	*Agrobacterium tumefaciens*	Nopaline synthase gene	338	81
