# Supplementary material for: Characterization of marine diatom-infecting virus promoters in the model diatom Phaeodactylum tricornutum
Source: Sci Rep. 2015 Dec 22;5:18708. doi: 10.1038/srep18708 (PMC4686930; doi:10.1038/srep18708)
Supplement: Supplementary Information [file srep18708-s1.doc]

**Supplementary Information**

**Characterization of marine diatom-infecting virus promoters in the model diatom**

***Phaeodactylum tricornutum***

Takashi Kadono^1^, Arisa Miyagawa-Yamaguchi^1,†^, Nozomu Kira^2^, Yuji Tomaru^3^, Takuma Okami^1^, Takamichi Yoshimatsu^1^, Liyuan Hou^4^, Takeshi Ohama^4^, Kazunari Fukunaga^1^, Masanori Okauchi^5^, Haruo Yamaguchi^1^, Kohei Ohnishi^6^, Angela Falciatore^7^ & Masao Adachi^1^

^1^ Laboratory of Aquatic Environmental Science, Faculty of Agriculture, Kochi University, Otsu-200, Monobe, Nankoku, Kochi 783-8502, Japan.

^2^ The United Graduate School of Agricultural Sciences, Ehime University, 3-5-7 Tarumi, Matsuyama, Ehime, 790-8566 Japan.

^3^ National Research Institute of Fisheries and Environment of Inland Sea, Fisheries Research Agency, 2-17-5 Maruishi, Hatsukaichi, Hiroshima 739-0452, Japan.

^4^ School of Environmental Science and Engineering, Kochi University of Technology, Tosayamada, Kami, Kochi 782-8502, Japan.

^5^ National Research Institute of Aquaculture, Fisheries Research Agency, 422-1 Nakatsuhamaura, Minami-ise, Mie 516-0193, Japan.

^6^ Research Institute of Molecular Genetics, Kochi University, Otsu-200, Nankoku, Kochi 783-8502, Japan.

^7^ Sorbonne Universités, UPMC Univ Paris 06, Institut de Biologie Paris-Seine, UMR 7238, F-75006 Paris, France; CNRS, UMR 7238, F-75006 Paris, France.

†Present address: Center for Innovative and Translational Medicine, Kochi University Medical School, Kohasu, Oko-cho, Nankoku, Kochi 783-8505, Japan.

Correspondence and requests for materials should be addressed to M.A. (email: madachi@kochi-u.ac.jp).

Here we provide Supplementary Tables 1−3, Supplementary Figures 1−8, Supplementary Data, and Supplementary References.

Supplementary Table 1 | Primers used in this study.

| Name | Sequence (5’-3’) | Annotation |
| --- | --- | --- |
| CdP1L/attB1 | GGGGACAAGTTTGTACAAAAAAGCAGGCTGCGGACCCATACCCCGA | add *att*B1 to the 5’ terminal of CdP1 |
| CdP1-2R/attB4 | GGGGACAACTTTGTATAGAAAAGTTGGGTTTTATTGTAAAATATGAAACGATTG | add *att*B4 to the 3’ terminal of CdP1 |
| CdP1(85-104)-F | GCGGTCCAGTGACGGTATTC | used for the genomic PCR analysis |
| ClP1L/attB1 | GGGGACAAGTTTGTACAAAAAAGCAGGCTTACGTAGAATCCTACGTAAA | add *att*B1 to the 5’ terminal of ClP1 |
| ClP1R/attB4 | GGGGACAACTTTGTATAGAAAAGTTGGGTGTGCGAATGTACGTGTTGT | add *att*B4 to the 3’ terminal of ClP1 |
| ClP1(84-104)-F | GAGTCCCGCCTGTTATTAACC | used for the genomic PCR analysis |
| ClP2L/attB1 | GGGGACAAGTTTGTACAAAAAAGCAGGCTTAGGAACATCCTTTGTGTTC | add *att*B1 to the 5’ terminal of ClP2 |
| ClP2R/attB4 | GGGGACAACTTTGTATAGAAAAGTTGGGTGAAGAACTTATTTTATGAC | add *att*B4 to the 3’ terminal of ClP2 |
| ClP2(121-140)-F | ATGTTGTATTGACCACGAGC | used for the genomic PCR analysis |
| TnitDNAV ORF1 Pro-(attB1)F | GGGGACAAGTTTGTACAAAAAAGCAGGCTTATACTTACTTACTGTTAGT | add *att*B1 to the 5’ terminal of TnP1 |
| TnitDNAV ORF1 Pro-(attB4)R | GGGGACAACTTTGTATAGAAAAGTTGGGTGATTTGTAAATTGTGGGAA | add *att*B4 to the 3’ terminal of TnP1 |
| TnitDNAV ORF2 Pro-(attB1)F | GGGGACAAGTTTGTACAAAAAAGCAGGCTTAATTTGTAAATTGTGGGAA | add *att*B1 to the 5’ terminal of TnP2 |
| TnitDNAV ORF2 Pro-(attB4)R | GGGGACAACTTTGTATAGAAAAGTTGGGTGTACTTACTTACTGTTAGT | add *att*B4 to the 3’ terminal of TnP2 |
| PtfcpA Pro-(attB1)F | GGGGACAAGTTTGTACAAAAAAGCAGGCTTAGGGCTGCAGGACGCAATGGAG | add *att*B1 to the 5’ terminal of PtfcpA pro. |
| PtfcpA Pro-(attB4)R | GGGGACAACTTTGTATAGAAAAGTTGGGTGTCTCGAAACGGCAGACAA | add *att*B4 to the 3’ terminal of PtfcpA pro. |
| CaMVPro/F/attB1 | GGGGACAAGTTTGTACAAAAAAGCAGGCTATGGTGGAGCACGACACT | add *att*B1 to the 5’ terminal of CaMV 35S pro. |
| CaMVPro/R/attB4 | GGGGACAACTTTGTATAGAAAATTGGGTGAGAGATAGATTTGTAGAG | add *att*B4 to the 3’ terminal of CaMV 35S pro. |
| CMVPro/F/attB1 | GGGGACAAGTTTGTACAAAAAAGCAGGCTTCAATATTGGCCATTAGC | add *att*B1 to the 5’ terminal of CMV pro. |
| CMVPro/R/attB4 | GGGGACAACTTTGTATAGAAAAGTTGGGTGGATCTGACGGTTCACTAA | add *att*B4 to the 3’ terminal of CMV pro. |
| nosPro/F/attB1 | GGGGACAAGTTTGTACAAAAAAGCAGGCTAACACTGATAGTTTAAAC | add *att*B1 to the 5’ terminal of *nos* pro. |
| nosPro/R/attB4 | GGGGACAACTTTGTATAGAAAAGTTGGGTGGCAGATTATTTGGATTGA | add *att*B4 to the 3’ terminal of *nos* pro. |
| CffcpPro(full)-F | GGATCCCTTTGTATTCAAAATAAGTATCG | used for the genomic PCR analysis |
| CffcpTer/F/attB3 | GGGGACAACTTTGTATAATAAAGTTGTGCGGCCGCATTGCTTGTTG | add *att*B3 to the 5’ terminal of CffcpA ter. for three-fragment Gateway® MultiSite recombination |
| CffcpTer/R/attB2 | GGGGACCACTTTGTACAAGAAAGCTGGGTGAGCTCTGGAAGCAT | add *att*B2 to the 3’ terminal of CffcpA ter. for two-/three-fragment Gateway® MultiSite recombination |
| CffcpTer/F/attB5 | GGGGACAACTTTGTATACAAAAGTTGTGTGCGGCCGCATTGCTTGTTG | add *att*B5 to the 3’ terminal of CffcpA ter. for two-fragment Gateway® MultiSite recombination |
| CffcpTer-R1 | GCAAGAAAACGGAAGGCTGG | used for the genomic PCR analysis |
| Cfnr Pro(416-436)-F | CGGTTCCCACGACTTGTTTGC | used for the genomic PCR analysis |
| TpfcpTer/F/attB3 | GGGGACAACTTTGTATAATAAAGTTGGCGCGGCCGCATACTGGATTG | add *att*B3 to the 5’ terminal of Tpfcp ter. for three-fragment Gateway® MultiSite recombination |
| TpfcpTer/R/attB2 | GGGGACCACTTTGTACAAGAAAGCTGGGTAGAGCTCCACCGCGGT | add *att*B2 to the 3’ terminal of Tpfcp ter. for two-/three-fragment Gateway® MultiSite recombination |
| TpfcpTer/F/attB5 | GGGGACAACTTTGTATACAAAAGTTGTGGCGCGGCCGCATACTGGATTG | add *att*B5 to the 3’ terminal of Tpfcp ter. for two-fragment Gateway® MultiSite recombination |
| ble/F/attB4r | GGGGACAACTTTTCTATACAAAGTTGATGGCCAAGTTGACCAGT | add *att*B4r to the 5’ terminal of *Sh ble* for three-fragment Gateway® MultiSite recombination |
| ble/R/attB3r | GGGGACAACTTTATTATACAAAGTTGTCAGTCCTGCTCCTCGGC | add *att*B3r to the 3’ terminal of *Sh ble* for three-fragment Gateway® MultiSite recombination |
| ble/F/attB1 | GGGGACAAGTTTGTACAAAAAAGCAGGCTATGGCCAAGTTGACCAGT | add *att*B1 to 5’ the terminal of *Sh ble* for two-fragment Gateway® MultiSite recombination |
| ble/R/attB5r | GGGGACAACTTTTGTATACAAAGTTGTTCAGTCCTGCTCCTCGGC | add *att*B5r to the 3’ terminal of *Sh ble* for two-fragment Gateway® MultiSite recombination |
| qPCRbleF3 | GGAGCGGTCGAGTTCTGGAC | detect *Sh ble* using qRT-PCR |
| qPCRbleR3 | GTGGACACGACCTCCGACCA | detect *Sh ble* using qRT-PCR |
| nat/F/attB4r | GGGGACAACTTTTCTATACAAAGTTGATGACCACTCTTGACGAC | add *att*B4r to the 5’ terminal of *nat* for three-fragment Gateway® MultiSite recombination |
| nat/R/attB3r | GGGGACAACTTTATTATACAAAGTTGTCAGGGGCAGGGCATGCT | add *att*B3r to the 3’ terminal of *nat* for three-fragment Gateway® MultiSite recombination |
| nat/F/attB1 | GGGGACAAGTTTGTACAAAAAAGCAGGCTATGACCACTCTTGACGAC | add *att*B1 to the 5’ terminal of *nat* for two-fragment Gateway® MultiSite recombination |
| nat/R/attB5r | GGGGACAACTTTTGTATACAAAGTTGTTCAGGGGCAGGGCATGCT | add *att*B5r to the 3’ terminal of *nat* for two-fragment Gateway® MultiSite recombination |
| Q-rps-fw | CGAAGTCAACCAGGAAACCAA | detect *rps* using qRT-PCR |
| Q-rps-rv | GTGCAAGAGACCGGACATACC | detect *rps* using qRT-PCR |
| eGFP/F/attB4r | GGGGACAACTTTTCTATACAAAGTTGCAATGGTGAGCAAGGGCGAG | add *att*B4r to the 5’ terminal of *egfp* for three-fragment Gateway® MultiSite recombination |
| eGFP/R/attB3r | GGGGACAACTTTATTATACAAAGTTGTTTACTTGTACAGCTCGTCC | add *att*B3r to the 3’ terminal of *egfp* for three-fragment Gateway® MultiSite recombination |
| EGFP-(attB1)F | GGGGACAAGTTTGTACAAAAAAGCAGGCTTAATGGTGAGCAAGGGCGAG | add *att*B1 to the 5’ terminal of *egfp* for two-fragment Gateway® MultiSite recombination |
| EGFP-(attB5r)R | GGGGACAACTTTTGTATACAAAGTTGTTGTTACTTGTACAGCTCGTCC | add *att*B5r to the 3’ terminal of *egfp* for two-fragment Gateway® MultiSite recombination |
| EGFP(qPCR)-F | TGCCCGACAACCACTACCTG | detect *egfp* using qRT-PCR |
| EGFP(qPCR)-R | CACGAACTCCAGCAGGACCA | detect *egfp* using qRT-PCR |

The underlined sections denote additional *att*B sequences used for the construction of the transformation vectors.

Supplementary Table 2 | Primers used in the genomic PCR analysis.

| Gene | Promoter | Forward primer | Reverse primer | Approximate size of PCR product (bp) |
| --- | --- | --- | --- | --- |
| *egfp* | Pro. less^*1^ | EGFP-(attB1)F | CffcpTer-R1 | 920 |
|  | PtfcpA pro. | PtfcpA Pro-(attB1)F | CffcpTer-R1 | 1370 |
|  | CdP1 | CdP1(85-104)-F | CffcpTer-R1 | 1300 |
|  | ClP1 | ClP1(84-104)-F | CffcpTer-R1 | 1350 |
|  | ClP2 | ClP2(121-140)-F | CffcpTer-R1 | 1280 |
|  | TnP1 | TnitDNAV ORF1 Pro-(attB1)F | CffcpTer-R1 | 1350 |
|  | TnP2 | TnitDNAV ORF2 Pro-(attB1)F | CffcpTer-R1 | 1350 |
|  | CaMV pro. | CaMVPro/F/attB1 | CffcpTer-R1 | 1380 |
|  | CMV pro. | CMVPro/F/attB1 | CffcpTer-R1 | 1670 |
|  | *nos* pro. | nosPro/F/attB1 | CffcpTer-R1 | 1270 |
| *Sh ble* | CffcpA pro. | CffcpPro(full)-F | CffcpTer-R1 | 2130 |
| *egfp* and *Sh ble*^*2^ | Cfnr pro. and CffcpA pro.^*2^ | Cfnr Pro(416-436)-F | CffcpTer-R1 | 3770 |

All primer sequences are shown in Supplementary Table 1. ^*1^ Pro. less indicates that no promoter is linked to the *egfp* gene of the transformation vector. ^*2^ For the transformation vector of pNICgfp^1^, we attempted to detect a single amplicon containing *egfp* driven by Cfnr pro. and *Sh ble* driven by CffcpA pro.

Supplementary Table 3 | Potential initiator-like (Inr-like) sequences in diatom endogenous and extrinsic viral promoters.

|  | Gene | Distance^*1^ | Potential Inr-like sequence | | | | | Accession number | Ref. |
| --- | --- | --- | --- | --- | --- | --- | --- | --- | --- |
|  |  |  | T | C | A | +1  H | W |  |  |
| Diatom | *Phaeodactylum tricornutum fcpC* | 48 | T | C | A | T | A | Z24768 | 2 |
|  | *Phaeodactylum tricornutum fcpD* | 48 | T | C | A | T | A | Z24768 | 2 |
|  | *Phaeodactylum tricornutum fcpE* | 26 | T | C | A | C | A | Z23153 | 2 |
|  | *Phaeodactylum tricornutum ca1* | 61 | T | C | A | C | A | AF414191 | 3 |
|  | *Cyclothella cryptica* *fcp2* | 2 | *T* | *C* | *A* | *A* | *A*^*2^ | AJ875044 | 4 |
|  |  | 23 | *T* | *C* | *A* | *A* | *T*^*2^ |  |  |
|  |  | 35 | *T* | *C* | *A* | *C* | *T*^*2^ |  |  |
|  | *Cyclothella cryptica* *fcp5* | 13 | *T* | *C* | *A* | *T* | *T*^*2^ | AJ875046 | 4 |
|  |  | 40 | *T* | *C* | *A* | *T* | *A*^*2^ |  |  |
|  | *Cyclothella cryptica* *fcp12* | 33 | *T* | *C* | *A* | *C* | *A*^*2^ | AJ875052 | 4 |
|  |  | 54 | *T* | *C* | *A* | *T* | *A*^*2^ |  |  |
|  | *Cyclothella cryptica* *fcp6* | 12 | *T* | *C* | *A* | *A* | *A*^*2^ | AJ875051 | 4 |
|  |  | 28 | *T* | *C* | *A* | *C* | *A*^*2^ |  |  |
|  |  | 44 | *T* | *C* | *A* | *C* | *A*^*2^ |  |  |
|  | *Cylindrotheca fusiformi*s *fcpA-1A* | 12 | *T* | *C* | *A* | *A* | *A*^*2^ | DQ060240 | 1 |
|  |  | 28 | *T* | *C* | *A* | *C* | *A*^*2^ |  |  |
|  |  | 42 | *T* | *C* | *A* | *T* | *A*^*2^ |  |  |
|  | *Cylindrotheca fusiformis* nitrate reductase gene | 60 | *T* | *C* | *A* | *A* | *T*^*2^ | DQ060241 | 1 |
|  | *Cylindrotheca fusiformis* α2 frustulin gene | 27 | *T* | *C* | *A* | *T* | *A*^*2^ | X99327 | 5 |
|  |  | 46 | *T* | *C* | *A* | *T* | *A*^*2^ |  |  |
| Virus | *Chaetoceros debilis* DNA virus putative replication-associated protein gene | 14  35 | *T*  *T* | *C*  *C* | *A*  *A* | *T*  *T* | *A*^*2^  *A*^*2^ | AB504376 | 6 |
|  | *Chaetoceros lorenzianus* DNA virus putative replication-associated protein gene | 36  61 | *T*  *T* | *C*  *C* | *A*  *A* | *T*  *T* | *A*^*2^  *A*^*2^ | AB553581 | 7 |
|  | *Chaetoceros lorenzianus* DNA virus putative structural protein gene | 40 | *T* | *C* | *A* | *T* | *A*^*2^ | AB553581 | 7 |
|  | *Thalassionema nitzschioides* DNA virus putative replication-associated protein gene | 35 | *T* | *C* | *A* | *T* | *T*^*2^ | AB781284 | 8 |
|  | *Thalassionema nitzschioides* DNA virus putative structural protein gene | 65 | *T* | *C* | *A* | *T* | *A*^*2^ |  | 8 |

^*1^ The distance shows the number upstream from the translational start codon to the +1 position in the Inr-like sequences. ^*2^ The Inr-like sequences shown in italics were identified via visual observations made within 80 bp from the translational start site, where core promoter motifs are typically located^9^.

Supplementary Figure 1

(Supplementary Figure 1; continued)

(Supplementary Figure 1; continued)

**Supplementary Figure 1 | Structure of potential viral promoters.** (a) Potential *cis*-regulatory elements and conserved motifs in the diatom-infecting virus (DIV) promoter regions. *Cis*-regulatory elements identified via the PLACE^10^ and PlantCARE^11^ programs shown here correspond to transcription factors (TFs) reported in the diatom genome^12^. For plant-type light-responsive *cis*-regulatory elements, all the sequences identified by the PlantCARE program are shown. The apex of a triangle on a line shows the location and direction of a *cis*-regulatory element. Here, right and left apexes show that a *cis*-regulatory element is located in the sense strand and anti-sense strand, respectively, positioned in the 5’-to-3’ direction on the locating strands. The diamonds show *cis*-regulatory elements that are located in both the sense and anti-sense strands. A green arrow above or below a line shows that a plant-type light-responsive *cis*-regulatory element is located in a sense or anti-sense strand, respectively. Conserved motifs (GGCAGGCG) were analysed using consensus motif-finding algorithms of CONSENSUS from Melina II^13^ using default parameters amongst potential DIV and extrinsic promoters (CaMV 35S, CMV, and *nos* promoters). The black arrows positioned above or below a line show that a conserved motif is located in a sense or anti-sense strand, respectively. The amplified region of ClP2 shows a lack of 25 bases from the translational start site (ATG). Inr: potential initiator-like sequences found in the diatom (further data are provided in the Discussion section and in Supplementary Table 3). (b) Sequence of the putative DIV promoter region. The black arrow above the line shows conserved motifs and *cis*-regulatory elements. Here, arrowheads on the right- and left-hand sides show that a conserved motif is located in the sense and anti-sense strands, respectively. A green arrow above or below a line shows that a plant-type light-responsive *cis*-regulatory element is found in the sense or anti-sense strand, respectively. The direction of the arrows show the location of *cis*-regulatory elements such as Myb, bZIP, CCAAT-binding, homeobox, and E2F-DP. The right and left arrows show that they are located in the sense strand and anti-sense strand, respectively. The two-directional arrows show that they are located in both the sense and anti-sense strands. The underlined area marks the primer annealing site. The red box shows potential core motifs such as the insect- and vertebrate-type homeobox binding sites detected via promoterome analysis^14^ (threshold > 95%).

Supplementary Figure 2

**Supplementary Figure 2 | Single-cassette vector for the transformation of *Phaeodactylum tricornutum* and *Chlamydomonas reinhardtii*.** The single-cassette vector for the transformation of *P. tricornutum* and *Chl. reinhardtii* contains the antibiotic-resistant gene *Sh ble*, which is controlled by various promoters used in this study. For the transformation of *P. tricornutum*, PtfcpA pro. and ClP1 were examined. For the transformation of *Chaetoceros* sp., CdP1 and ClP1 were examined. For the transformation of *Chl. reinhardtii* CC-503, all the tested promoters used in the double-cassette vector were examined. CffcpA ter.: terminator region of the fucoxanthin chlorophyll *a*/*c*-binding protein A-1A gene derived from *Cylindrotheca fusiformis*. *Sh ble*: the bleomycin-resistant gene derived from *Streptoalloteichus hindustanus*.

Supplementary Figure 3

**Supplementary Figure 3 | Single-cassette vectors for the transformation of Centric diatoms.** Vectors contain the antibiotic gene *nat* controlled by the DIV promoter (CdP1 and ClP1). Tpfcp ter.: terminator region of the fucoxanthin chlorophyll *a*/*c*-binding protein gene derived from *Thalassiosira pseudonana*. *nat*: the nourseothricin-resistant gene derived from *Streptomyces noursei*.

Supplementary Figure 4

**Supplementary Figure 4 | Annealing sites of the primers used for the genomic PCR (a) and the results of the genomic PCR analysis of the transformed *P. tricornutum* (b).** Genomic PCR amplifications of the tested pro.-*egfp* with the forward primer of the tested promoter or with EGFP-(attB1)F and CffcpTer-R1, CffcpA pro.-*Sh ble* with CffcpPro(full)-F, and CffcpTer-R1 using the *P. tricornutum* transformant cells as templates were performed, and the amplified products were electrophoresed. WT represents the wild type cells as a negative control. The primers used for the genomic PCR analysis are shown in Supplementary Tables 1 and 2. M: DNA size marker.

Supplementary Figure 5

**Supplementary Figure 5 | The transformation vector pNICgfp with annealing sites of the primers used for genomic PCR (a) and the results of the genomic PCR analysis of the transformed *P. tricornutum* (b).** Genomic PCR amplification with a forward primer, Cfnr Pro(416-436)-F, and a reverse primer, CffcpTer-R1, using the *P. tricornutum* transformant cells as templates was performed, and the amplified products were electrophoresed. WT represents the wild type cells as a negative control. The primers used for the genomic PCR analysis are shown in Supplementary Tables 1 and 2. M: DNA size marker. The vector map of pNICgfp^1^ was modified from Miyagwa *et al*.^15^.

Supplementary Figure 6

(Supplementary Figure 6; continued)

**Supplementary Figure 6 | Variations in the expression levels of transgenes driven by potential promoters from DIVs in ten transformants.** Ten independent transformants for each promoter were analysed. The left scale indicates the expression levels of *egfp* and *Sh ble* normalized to an internal control gene (ribosomal protein small subunit 30S gene, *rps*). The right-hand scale indicates the ratios of the expression level of *egfp* to that of *Sh ble*.

Supplementary Figure 7

**Supplementary Figure 7 | Abundance of eGFP protein translated from transcripts driven by various promoters in transformants using flow cytometry.** Ten independent transformants derived from various promoters with the exception of the CaMV 35S promoter were analysed. For the CaMV 35S promoter, nine independent transformants were analysed. The circles denote the mean value for approximately 10,000 cells of independent transformants. The diamonds denote the average values of the transformants. Asterisks indicate statistically significant differences derived from the PtfcpA pro. transformants (***P* < 0.01 and **P* < 0.05). The broken line denotes the autofluorescence level of wild type cells excited at 488 nm.

Supplementary Figure 8

**Supplementary Figure 8 |** Potential *cis*-regulatory elements and conserved motifs in the CaMV 35S promoter, the CMV promoter, and the *nos* promoter. *Cis*-regulatory elements identified via the PLACE^10^ and PlantCARE^11^ programs shown here correspond to transcription factors (TFs) reported in the diatom genome^12^. For plant-type light-responsive *cis*-regulatory elements, all the sequences identified by the PlantCARE program are shown. The apex of a triangle on a line shows the location and direction of a *cis*-regulatory element. Here, right and left apexes show that a *cis*-regulatory element is located in the sense strand and anti-sense strand, respectively, positioned in the 5’-to-3’ direction on the locating strands. The diamonds show *cis*-regulatory elements that are located in both the sense and anti-sense strands. A green arrow above or below a line shows that a plant-type light-responsive *cis*-regulatory element is located in a sense or anti-sense strand, respectively. Conserved motifs (GGCAGGCG) were analysed using consensus motif-finding algorithms of CONSENSUS from Melina II^13^ using default parameters amongst potential DIV and extrinsic promoters (CaMV 35S, CMV, and *nos* promoters). The black arrows positioned above or below a line show that a conserved motif is located in a sense or anti-sense strand, respectively. Inr: potential initiator-like sequences found in the diatom (further data are provided in the Discussion section and in Supplementary Table 3).

**Supplementary Data**

Output data obtained from the transcription factor binding site (TFBS) cluster analysis (TCA) conducted in oPOSSUM version 3^16^, showing that the locations of “Conserved C113 Binding Sites” include insect- and vertebrate-type homeobox binding sites. In Supplementary Figure 1, we show the sequences with thresholds exceeding 95%.

**Supplementary References**

1. Poulsen, N. & Kröger, N. A new molecular tool for transgenic diatoms: control of mRNA and protein biosynthesis by an inducible promoter-terminator cassette. *FEBS J.* **272**, 3413−3423 (2005).

2. Bhaya, D. & Grossman, A. R. Characterization of gene clusters encoding the fucoxanthin chlorophyll proteins of the diatom *Phaeodactylum tricornutum*. *Nucleic Acids Res.* **21**, 4458−4466 (1993)

3. Harada, H., Nakatsuma, D., Ishida, M. & Matsuda, Y. Regulation of the expression of intracellular beta-carbonic anhydrase in response to CO_2_ and light in the marine diatom *Phaeodactylum tricornutum*. *Plant Physiol.* **139**, 1041−1050 (2005).

4. Brakemann, T., Frank, B., Peter, K. & Erhard, R. Structural and functional characterization of putative regulatory DNA sequences of *fcp* genes in the centric diatom *Cyclotella cryptica*. *Diatom Res.* **23**, 31−49 (2008).

5. Kröger, N., Bergsdorf, C. & Sumper, M. Frustulins: domain conservation in a protein family associated with diatom cell walls. *Eur. J. Biochem.* **239**, 259−264 (1996).

6. Tomaru, Y., Shirai, Y., Suzuki, H., Nagasaki, T. & Nagumo, T. Isolation and characterization of a new single-stranded DNA virus infecting the cosmopolitan marine diatom *Chaetoceros debilis*. *Aquat. Microb. Ecol.* **50**, 103−112 (2008).

7. Tomaru, Y. *et al*. Isolation and characterization of a single-stranded DNA virus infecting *Chaetoceros lorenzianus* Grunow. *Appl. Environ. Microbio.l* **77**, 5285−5293 (2011).

8. Tomaru, Y. *et al*. First evidence for the existence of pennate diatom viruses. *ISME J.* **6**, 1445−1448 (2012).

9. Juven-Gershon, T. & Kadonaga, J. T. Regulation of gene expression via the core promoter and the basal transcriptional machinery. *Dev. Biol.* **339**, 225−229 (2010).

10. Higo, K., Ugawa, Y., Iwamoto, M. & Korenaga, T. Plant cis-acting regulatory DNA elements (PLACE) database: 1999. *Nucleic Acids Res.* **27**, 297−300 (1999).

11. Lescot M*, et al.* PlantCARE, a database of plant cis-acting regulatory elements and a portal to tools for *in silico* analysis of promoter sequences. *Nucleic Acids Res.* **30**, 325−327 (2002).

12. Rayko, E., Maumus, F., Maheswari, U., Jabbari, K. & Bowler, C. Transcription factor families inferred from genome sequences of photosynthetic stramenopiles. *New Phytol.* **188**, 52−66 (2010).

13. Okumura, T., Makiguchi, H., Makita, Y., Yamashita, R. & Nakai, K. Melina II: a web tool for comparisons among several predictive algorithms to find potential motifs from promoter regions. *Nucleic Acids Res.* **35**, W227−231 (2007).

14. Russo, M. T., Annunziata, R., Sanges, R., Ferrante, M. I. & Falciatore, A. The upstream regulatory sequence of the light harvesting complex *Lhcf2* gene of the marine diatom *Phaeodactylum tricornutum* enhances transcription in an orientation- and distance-independent fashion. *Mar. Genomics* doi: 10.1016/j.margen.2015.06.010 (2015).

15. Miyagawa, A. *et al*. Research note: High efficiency transformation of the diatom *Phaeodactylum tricornutum* with a promoter from the diatom *Cylindrotheca fusiformis*. *Phycological Res.* **57**, 142−146 (2009).

16. Kwon, A. T., Arenillas, D. J., Worsley Hunt, R., Wasserman, W. W. oPOSSUM-3: advanced analysis of regulatory motif over-representation across genes or ChIP-Seq datasets. *G3 (Bethesda)* **2**, 987−1002 (2012).
